# Retino‐cortical stimulus frequency‐dependent gamma coupling: evidence and functional implications of oscillatory potentials

**DOI:** 10.14814/phy2.12986

**Published:** 2016-10-04

**Authors:** Mihail I. Todorov, Katalin A. Kékesi, Zsolt Borhegyi, Robert Galambos, Gábor Juhász, Anthony G. Hudetz

**Affiliations:** ^1^ Laboratory of ProteomicsInstitute of BiologyEötvös Loránd UniversityBudapestHungary; ^2^ Department of Physiology and NeurobiologyEötvös Loránd UniversityBudapestHungary; ^3^ MTA‐ELTE‐NAP B‐Opto‐Neuropharmacology GroupEötvös Loránd UniversityBudapestHungary; ^4^ University of California San DiegoLa JollaCalifornia; ^5^ Department of AnesthesiologyCenter for Consciousness ScienceUniversity of MichiganAnn ArborMichigan

**Keywords:** Oscillations, perception, synchrony

## Abstract

Long‐range gamma band EEG oscillations mediate information transmission between distant brain regions. Gamma band‐based coupling may not be restricted to cortex‐to‐cortex communication but may include extracortical parts of the visual system. The retinogram and visual event‐related evoked potentials exhibit time‐locked, forward propagating oscillations that are candidates of gamma oscillatory coupling between the retina and the visual cortex. In this study, we tested if this gamma coupling is present as indicated by the coherence of gamma‐range (70–200 Hz) oscillatory potentials (OPs) recorded simultaneously from the retina and the primary visual cortex in freely moving, adult rats. We found significant retino‐cortical OP coherence in a wide range of stimulus duration (0.01–1000 msec), stimulus intensity (800–5000 mcd/mm^2^), interstimulus interval (10–400 msec), and stimulus frequency (0.25–25 Hz). However, at low stimulus frequencies, the OPs were time‐locked, flickering light at 25 Hz entrained continuous OP coherence (steady‐state response, SSR). Our results suggest that the retina and the visual cortex exhibit oscillatory coupling at high‐gamma frequency with precise time locking and synchronization of information transfer from the retina to the visual cortex, similar to cortico‐cortical gamma coupling. The temporal fusion of retino‐cortical gamma coherence at stimulus rates of theater movies may explain the mechanism of the visual illusion of continuity. How visual perception depends on early transformations of ascending sensory information is incompletely understood. By simultaneous measurement of flash‐evoked potentials in the retina and the visual cortex in awake, freely moving rats, we demonstrate for the first time that time‐locked gamma oscillatory potentials exhibit stable retino‐cortical synchrony across a wide range of stimulus parameters and that the temporal continuity of coherence changes with stimulus frequency according to the expected change in the visual illusion of continuity.

## Introduction

How the conscious visual experience emerges from neuronal spiking and field potential activities has been an intriguing but elusive problem of neuroscience (Wachtmeister 1986). Melloni et al. (2009) theorized that organized local field potential oscillations along the hierarchy of the visual system play a critical role in conscious visual perception. In the retina, a high‐frequency (70–200 Hz) oscillatory potential (OP) is evoked by a single flash of light. The OP is a special type of a transient and millisecond‐precise package that rides on the ascending limb of the electroretinogram (ERG) and its frequency matches the EEG gamma waves, hypothesized as a long‐range coupling signal. The retinal OP signal was discovered by Fröhlich (1914), but its function in the transfer of visual information is still unclear. An oscillatory component of the visual evoked potentials (VEP) that resembles the retinal OP in frequency and timing has been independently described in the primary visual cortex (V1) (Lopez et al. 2002; Rajkai et al. 2008). The relationship between these two gamma‐band oscillatory potentials (called *omega*‐oscillations) was characterized partly by Munk and Neuenschwander (2000). While cortico‐cortical gamma frequency coupling and its role in cognitive function have been extensively investigated (Buzsaki and Wang 2012), the retinal and V1 oscillatory potentials attracted little attention as functional coupling signals. Although in clinical applications, the two signals have been used as independent diagnostic signals as ERG and VEP due to the lack of direct evidence for a functional link between the retinal OP and V1.

Retinal OPs were traditionally considered as local oscillations triggered by a flash onset (Dong et al. 2004). In contrast, V1 gamma range oscillations are considered as a part of the cortical gamma activity generated cooperatively by the incoming visual activity and by the local gamma oscillation mechanisms based on reciprocal activation of pyramidal cells and local interneurons. While the oscillation‐based coupling between the lateral geniculate nucleus (LGN) and the V1 is well established (Bekisz and Wrobel 1999), this has not been implicated for a coupling between the retina and the V1. The mechanism of information transfer between the retina and the LGN may rely on the S potential discovered by Kaplan and Shapley (1984). The S potential is the presynaptic optic nerve potential at the LGN neurons and its correlation with the LGN cell firing suggests a spike‐to‐spike type transmission from the retina to the LGN. Therefore, it is legitimate to hypothesize a similar oscillation‐based coupling between the retina and the V1 via the optic nerve that is relayed to the cortex by the LGN.

The generation and recording of field potentials in the retina is different from those in the brain. During normal visual experience, the small number of neurons in the thin retinal layers does not generate spontaneous field potential fluctuations large enough, like the cortical EEG. Retinal OP components become evident in flash‐evoked field potentials when the flash induces hypersynchronous activity in all retinal elements. Also, the retina generates two types of stimulus related potentials depending on the stimulating flash frequency. One is the single flash response or classical ERG discovered by Granit (1933). The other one is the entrained steady‐state response (SSR) evoked by continuous application of flashes at a rate above 20 Hz (Beverina et al. 2003), thus in the retina, we are limited to two types of evoked potentials. To examine the retino‐cortical coupling of OPs, we need a direct evidence for the spreading of OPs from the retina to the V1 at various stimulus parameters in conscious animals. We formerly described a rat implant (Szabo‐Salfay et al. 2001) to record flash‐evoked responses at the retina, optic chiasm, and V1 in freely moving animals. In that model, the flashes from the retrobulbarly placed LED provided a fast, controllable, and monochromatic light free from changes in the number of photons due to the lens and pupillary dilatation thus being well suited to investigate the eye–V1 gamma coupling in different stimulus conditions.

## Methods

### Surgical methods

All animal experiments were done under the local ethical rules that are in accordance with the EU regulation for use animals for scientific experiments (2010/63/EU revising Directive 86/609/EEC on the protection of animals used for scientific purposes). We applied advanced anesthesia and pain relief in order to minimize the suffering and pain of the animals. The protocol of rat implantation has been described earlier in details (Szabo‐Salfay et al. 2001). Briefly, adult Sprague–Dawley (Charles River Laboratories, Toxicoop Hunagry, Ltd., Budapest, Hungary) and Wistar rats over 250 g were implanted in Halothane air mixture (1% Halothane in air). Rats were placed into a stereotaxic frame. A stimulating LED (5 mm, high emission, BI‐B6334SQD, Bright LED Electronics, Hong Kong, China) and a background light LED (3 mm red body, low‐emission LED) were placed retrobulbarly above the left eye. An eye ball electrode, a multistrand stainless steel wire (Medwire 7SST, Medwire Corporation, Mt. Vernon, NY), was placed to the surface of the eyeball under the eyelids. The optic chiasm was ipsilaterally recorded by an electrolytically edged tungsten wire electrode insulated by EpoxyLite. Coordinates for implantation: A: −1.1; L: 1.1; V: 9.8. For accurate vertical positioning, we gently touched the bone with the electrode tip, and then pulled it up 0.2 mm. Stainless screw electrodes of 0.8 mm OD were placed above the primary visual cortex areas at both sides to record cortical evoked potentials. A stainless steel plate implanted under the skin of the head of the rats was used as a reference electrode and grounding was made through a screw electrode placed above the cerebellum. Electrodes were soldered to a socket and a flexible wire lead connected them to a Grass 8B EEG amplifier system (Grass Instruments, West Warwick, RI). LEDs were connected to a separate socket to avoid stimulus artifact.

### Stimulation with LED

Red colored LEDs were used to avoid the filtering effect of hemoglobin. LED light emission increased exponentially by current flowing through the diode. At the saturation current, however, the number of emitted photons cannot be increased by current and the energy excess dissipates as heat. Therefore, we used a LED adaptor (a regulated current source) adjusting the current on the LED always slightly above saturation current to get a reproducible flash but avoiding heat. The adaptor was connected to a digital stimulator (Supertech Co, Budapest, Hungary), and the duration, frequency, and amount of flashes were adjusted by the stimulator. The minimum duration was limited to 0.01 msec. For luminance series, we used a standard resistor series to divide the saturation current to 6 reproducible luminance levels calibrated by a lux meter (800, 1600, 2500, 3300, 4100, 5000 mcd).

The recording room was dark and the light intensity was less than 0.017 lux measured by a lux meter. Dark adaptation was 30 min before recording. In single flash mode, we delivered flashes every 5 sec (0.25 Hz) because we found no interaction of flashes at that low‐frequency range in a former pilot study. In flickering light mode, however, (3, 9, 11, 25 Hz), the flickering light itself produced light adaptation after a few flickers. The initial response is a dark adapted one but later the SSRs are light‐adapted responses. The recording paradigm of SSRs was so that we set a 1 min pause between two 30 sec duration series of flashes. So the light adaptation levels were kept standard in SSR experiments.

### Data acquisition and analysis

Electrophysiological recording was done using a Grass 8B EEG machine in a frequency band from 0.1 Hz to 10 kHz. The calibration signal was 50 μV. Responses were collected by a CED 1401 data acquisition system using Signal 1.93 software. The sampling rate was 5 kHz. In all experiments, we used single frame recording mode, and before averaging, we sorted out the frames containing motor artifacts manually. An average of 100 responses was used in statistical analysis and gamma filtering. Averaging was performed offline by the CED Signal 1.93 software and gamma filtering was made by a FFT high pass filter option of Origin 8.5 data processing and scientific graphics software. The FFT filter was adjusted to 70 Hz high‐pass to separate gamma oscillations.

Gamma filtering was made by a second‐order, high‐pass Bessel filter at 70 Hz cut‐off frequency. Data analysis was performed in MATLAB 2013, using scripts of EEGLAB ERPLAB open access software (Delorme and Makeig 2004). In data processing protocol, we did gamma filtering from the averaged responses (*n* = 40 in an average) and performed the Morlet wavelet analysis to show gamma activity in the cortex, optic chiasm and retinal responses, data analysis, power coherence correlation phase relations. Then, we calculated retino‐cortical coherence for demonstrating the frequency and timing of coherence, more details are in the Supplementary Materials.

### TTX application on V1 surface

In three rats, we implanted a guide cannula closely attached to the screw electrode during the initial surgery. A microliter Hamilton syringe was equipped with a blocker to stop the syringe tip when it is on the cortical surface. Hence, we were able to make a local injection of TTX to the surface of V1. The freely moving rats were let to recover for 5 days, and then cortical and retinal evoked potentials to 0.25 Hz red LED flashes were recorded. First, we recorded control responses to 1 μL saline injection, then we injected 1 μL, 1 μmol/L TTX containing saline, and repeated the recording 10 min after the injection.

### Statistics

Data analysis was performed using OriginPro (OriginLab Corporation, Northampton, MA) software's Descriptive Statistics algorithm. The latencies of the OPs were measured as the time between the stimulus and the peak of the OP component (OP1, OP2, OP3, and OP4). Measurements are expressed as the mean ± SD.

## Results

### Single‐flash response OPs

**Figure 1:**
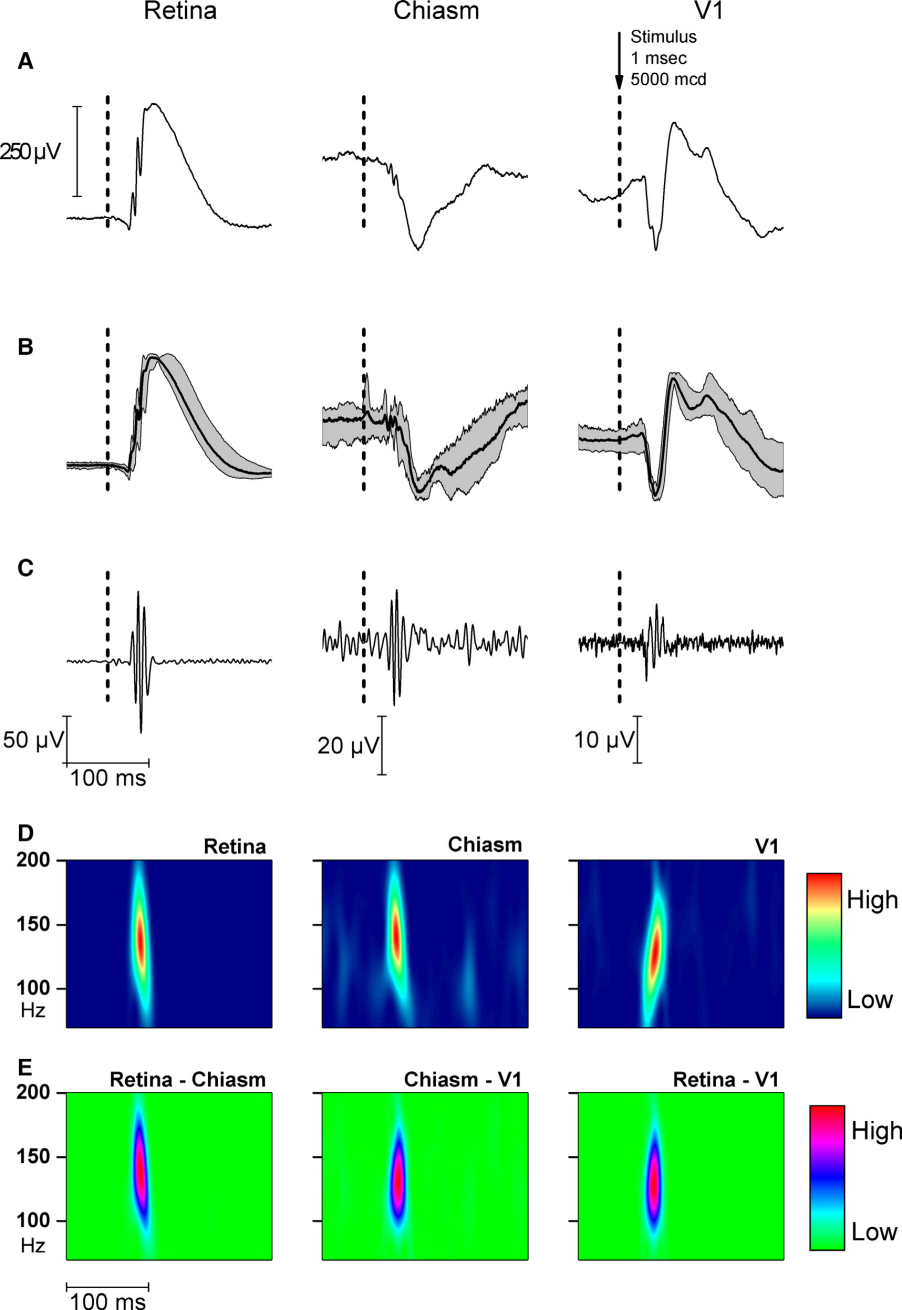
Single‐flash responses recorded from the retina, optic chiasm, and V1. (A) Average of 25 sweeps from one representative animal. (B) Grand average of the retina, optic chiasm, and V1 responses from five animals, showing confidence intervals and mean values. (C) Filtered oscillatory potential responses from one animal (Bessel filter 70–200 Hz). (D) Wavelet spectra of responses from 70 to 250 Hz frequency band. (E) Frequency cross‐coherence between the retina‐chiasm OPs (left), the chiasm‐V1 OPs (middle), and the retina‐V1 (right).

**Figure 2:**
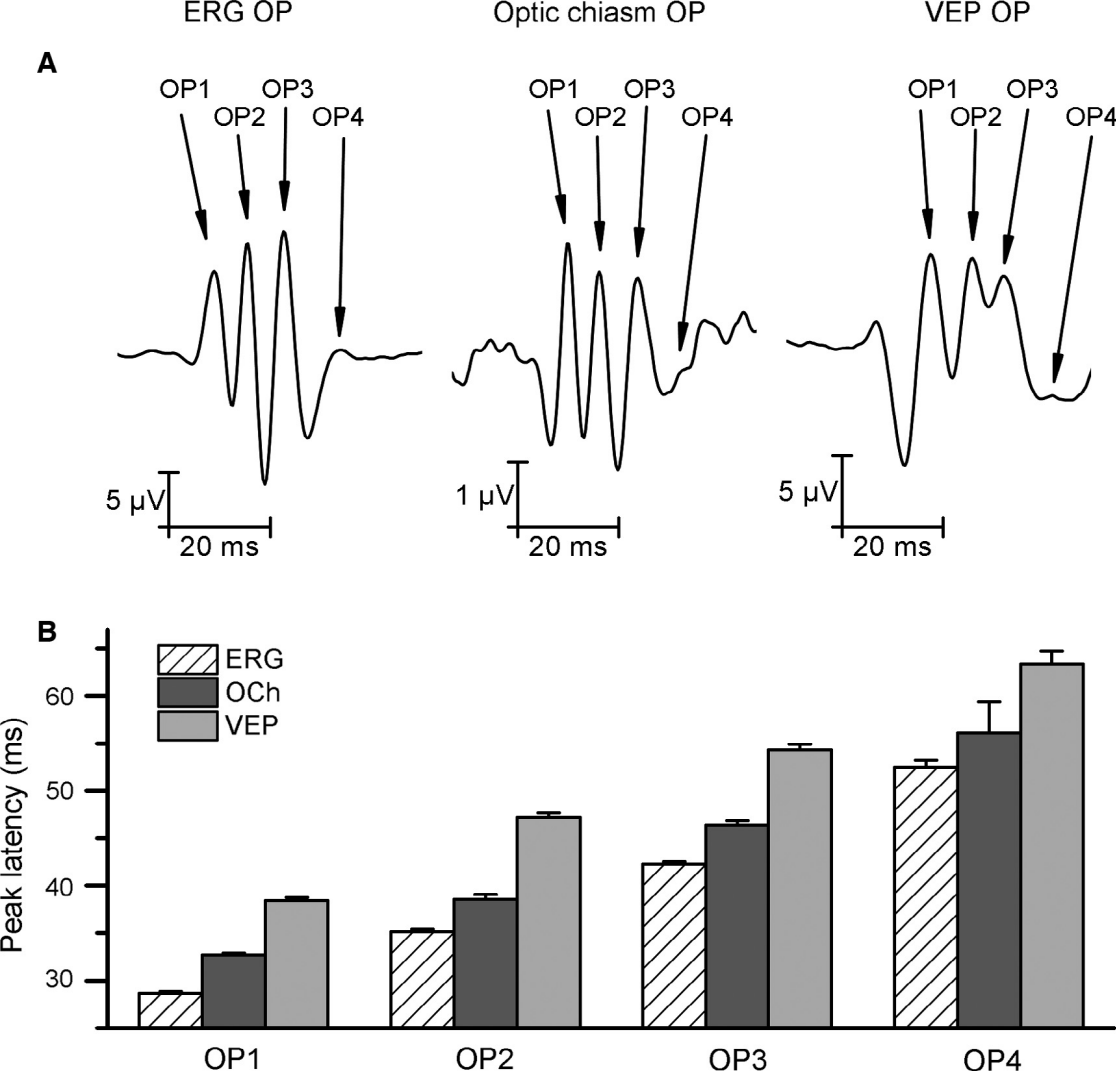
Four components of oscillatory potentials (OP) recorded from the retina (ERG), chiasm (RFU), and visual cortex V1 (VEP) from all animals.(A) Waveform of normalized, filtered, averaged (*n* = 50) OP responses to single 1 msec flash. The four components of OPs are indicated. (B) Peak latency of the four OP components. Note the delay of each component of the OPs recorded at the beginning, middle and at the end of the visual pathway indicating that OPs generated in the retina spread to V1 through the optic nerve. Data are mean ± SD.

Stimulating the retina with 1 msec duration, randomly applied flashes at mean frequency lower than 0.25 Hz evoked ERG, chiasm ERP, and VEP (Fig. 1A). The responses were similar in five rats as indicated by the confidence intervals of grand averages (Fig. 1B). Applying a 70 Hz high‐pass filter, gamma‐frequency component of OPs could be separated from the traces (Fig. 1C). The wavelet transform of the signals revealed time‐locked gamma oscillation components in ERG, chiasm ERP, and VEP, the high‐frequency (100–200 Hz) range (Fig. 1D). In the cortex, we obtained a slower, 125 Hz oscillation at high power. Coherence between the retina and V1 was a strong single frequency coherence at mean of 120 Hz. The coherence pattern of the chiasm and retina as well as chiasm and V1 were more complex (Fig. 1E). The stimulus onset‐locked gamma oscillations were very similar in all recorded areas of the visual system. Superimposing the normalized OPs of the retina, chiasm, and V1 revealed a phase shift from the eye to the cortex implying that the OPs in the chiasm and the V1 were not the result of volume conduction (Fig. 2A). The phase shift of each OP volley was different, the first component had the shortest retino‐cortical phase shift and the last one had the longest one (Fig. 2B).

**Figure 3:**
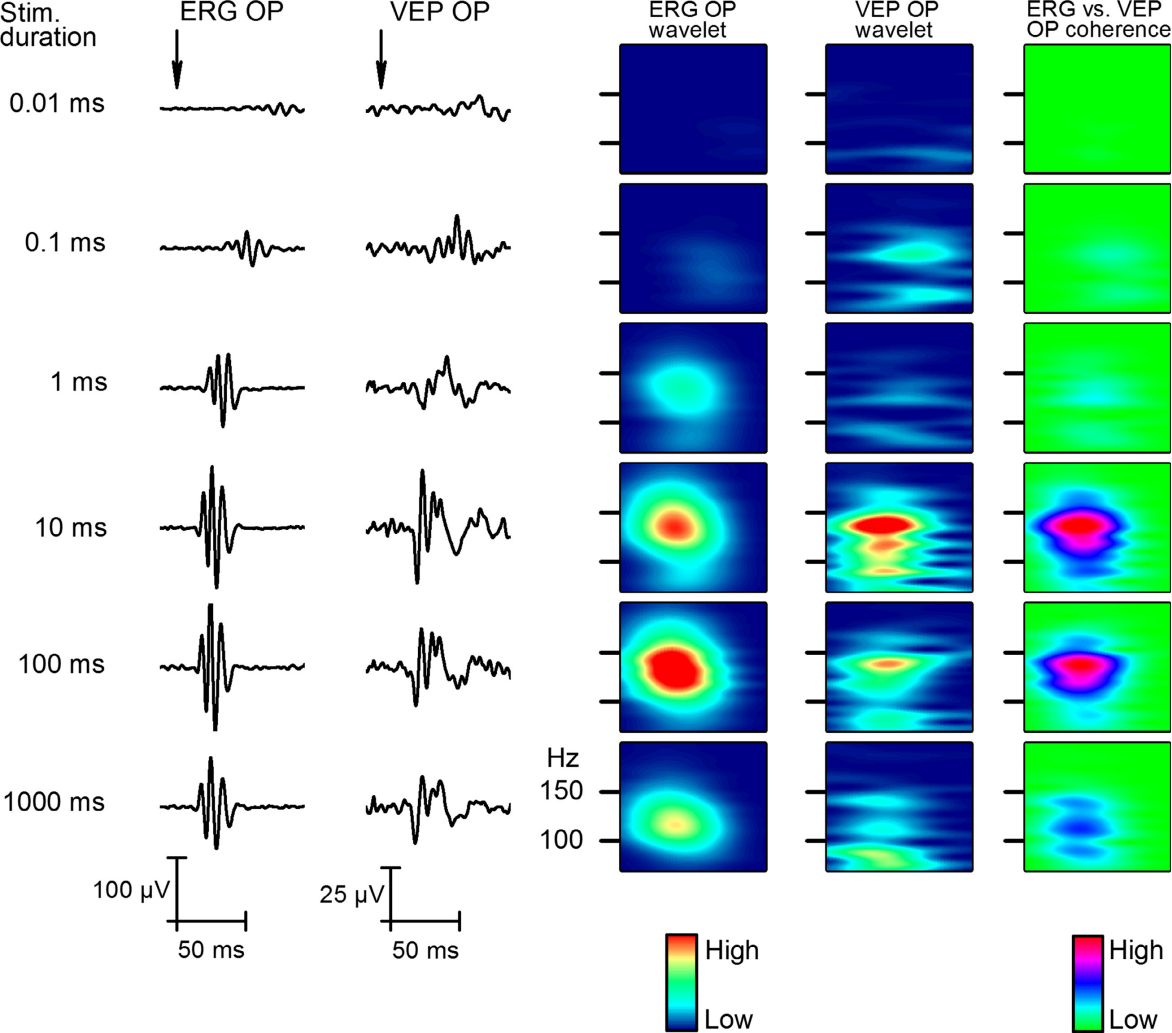
Results of flash duration experiments. Two columns on the left show averaged (*n* = 50) responses recorded from the cornea and V1 to the 0.01–1000 msec stimulus length. The next two columns present the results in form of wavelets of the filtered signal (70–200 Hz, Bessel). The last column indicates the coherence between the cornea and V1 signal.

**Figure 4:**
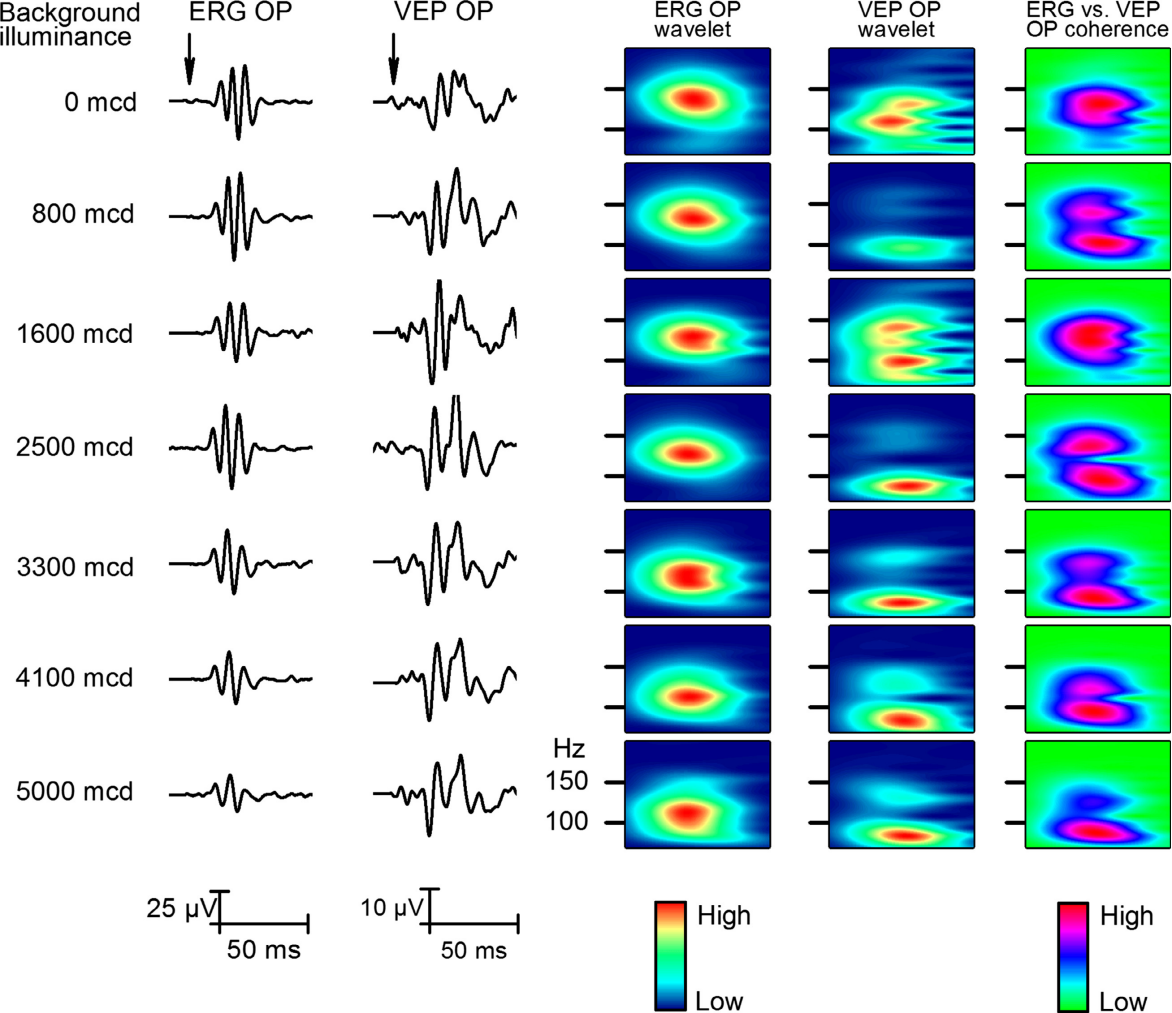
Effect of background luminance on the retinal and V1 responses. Two columns on the left show changes in averaged responses (*n* = 50) to seven different background illumination level intensities from 0 to 5000 mcd. The next two columns show wavelets of the filtered signal (70–200 Hz, Bessel). The last column shows retina‐V1 coherence analysis of OPs.

To determine how the OPs of the retina and the cortex responded to the change in stimulus duration, we changed it from 0.01 to 1000 msec. The stimulus duration series were recorded from five animals and Figure 3 shows that only the very short flashes (0.01–1 msec) induced different oscillations; from 10 to 1000 msec, the OPs remained the same. On the retino‐cortical coherence plot (Fig. 3C), we also demonstrated that the phase coherence of retinal and cortical OPs does not reflect the stimulus length from 10 to 1000 msec flash duration range. Szabo‐Salfay et al. (2001) showed that visual evoked responses are influenced by the background light. Implanting a background light, LED together with the stimulating LED, we applied four different intensity background light intensities adjusted by a resistor series connected in line with the background light LED. The adaptation light intensities measured before implantation were 0, 800, 1600, 2500, 3300, 4100, and 5000 mcd. The wavelet and phase coherence analysis of the data (Fig. 4B and C, respectively) shows that only the highest adaptation level (5000 mcd) changed the wavelet results and retino‐cortical OP coherence pattern. We concluded that the background illumination intensity did not influence the OPs in the retina and V1.

### The effect of interstimulus interval

**Figure 5:**
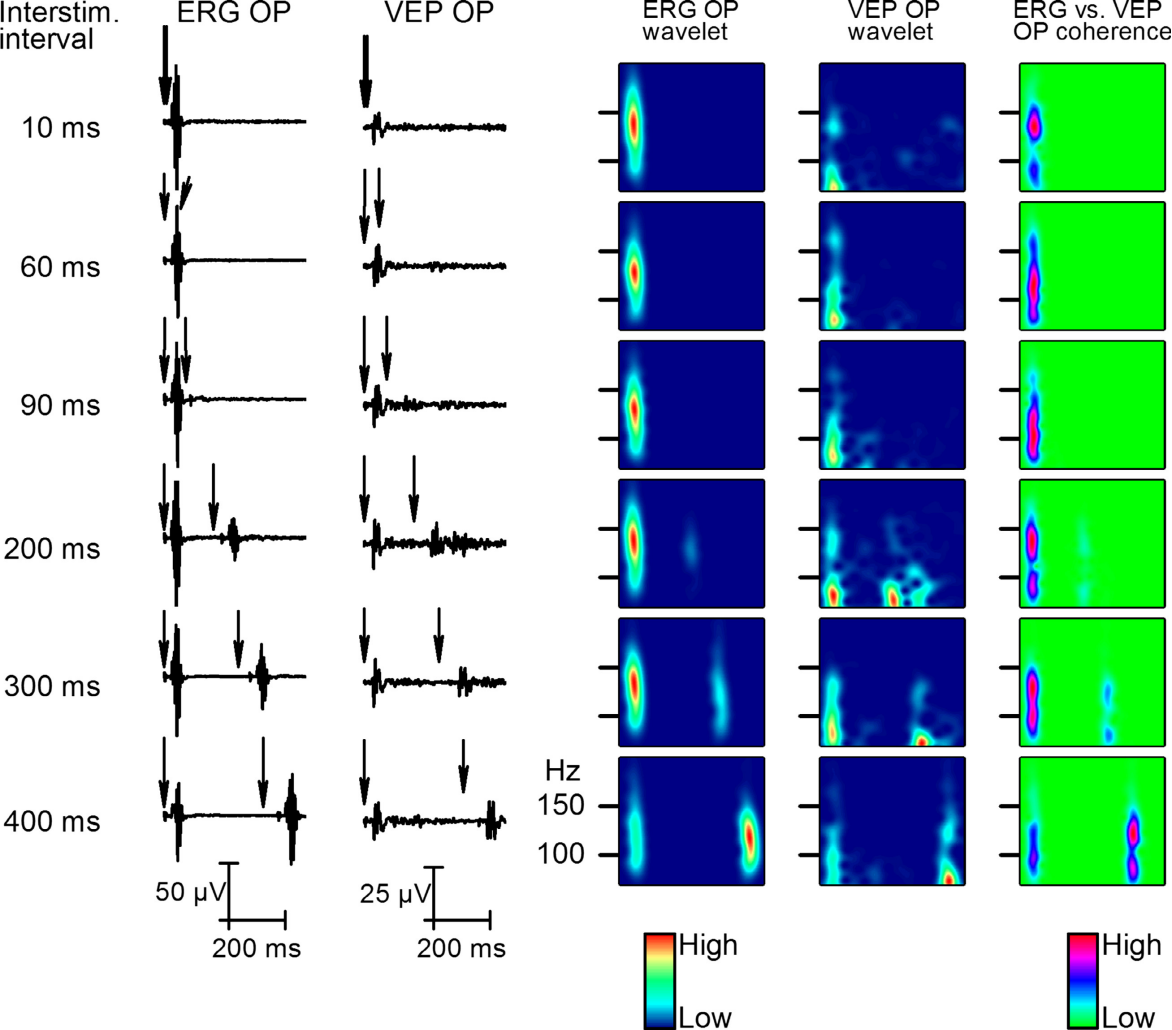
Effect of increasing interstimulus (ISI) interval on the second response. The two columns on the left show changes in averaged responses (*n* = 50) to six different ISIs from 10 to 400 msec. The next two columns show the wavelet analysis results of OPs filtered form the retina and V1. The column on the right indicates coherence of the retina and V1 response OPs at different ISIs.

The recovery period of the visual system after a flash can be tested by an interstimulus interval (ISI) series that may provide information on how the system is capable to analyze the next flash. We applied pairs of 1 msec flashes (S1 and S2) with different ISIs (Fig. 5). The OP response to the second flash appeared at 200 msec ISI and it required 400 msec for full recovery. The S2 OPs were not present up to 90 msec ISIs (Fig. 5A) in spite of the observation that a human experimenter was able to see two distinct light flashes on the animal's head at ISI intervals even at less than 100 msec. The wavelet analysis showed that the OPs appear on S2 at 200 msec ISI but the OPs of S2 were not coherent between the retina and cortex. The OPs of S1 in retina and V1 responses were in solid coherence independently of the coherence of the second response at all ISI length studied (Fig. 5A–C). At 300 and 400 msec ISI, the retinal and V1 OPs became coherent (Fig. 5C).

### Effect of stimulus frequency

**Figure 6:**
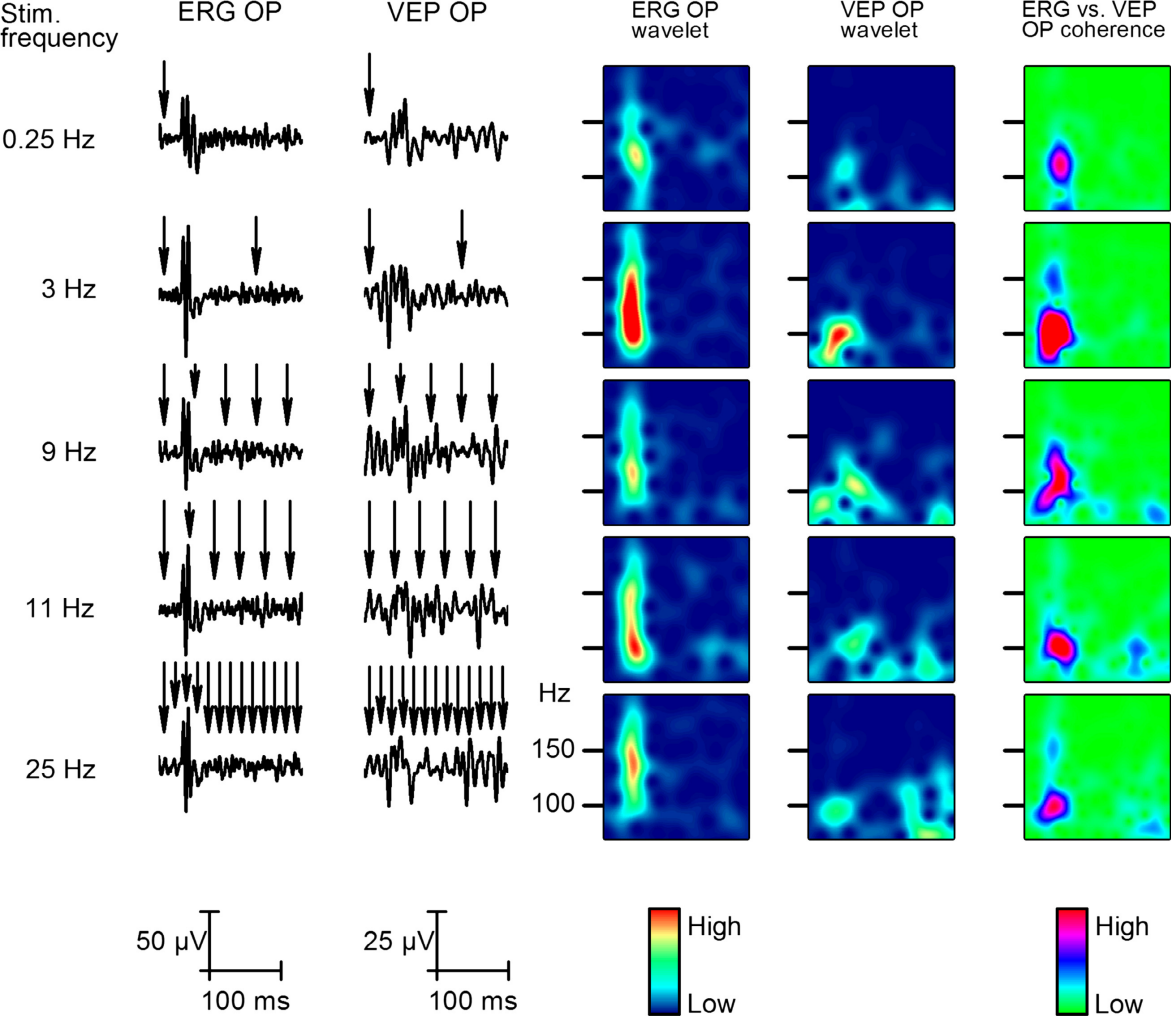
Effect of stimulus frequency on the early response. The two columns on the left show changes in averaged early steady‐state responses (SSR, 0–200 msec after stimulus start, *n* = 50) to five different stimulus frequencies from 0.25 to 25 Hz. The next two columns show the wavelet analysis results of early SSR OPs recorded from the retina and V1. The column on the right indicates coherence between the retina and V1 at the early stage SSR OPs at different frequencies.

**Figure 7:**
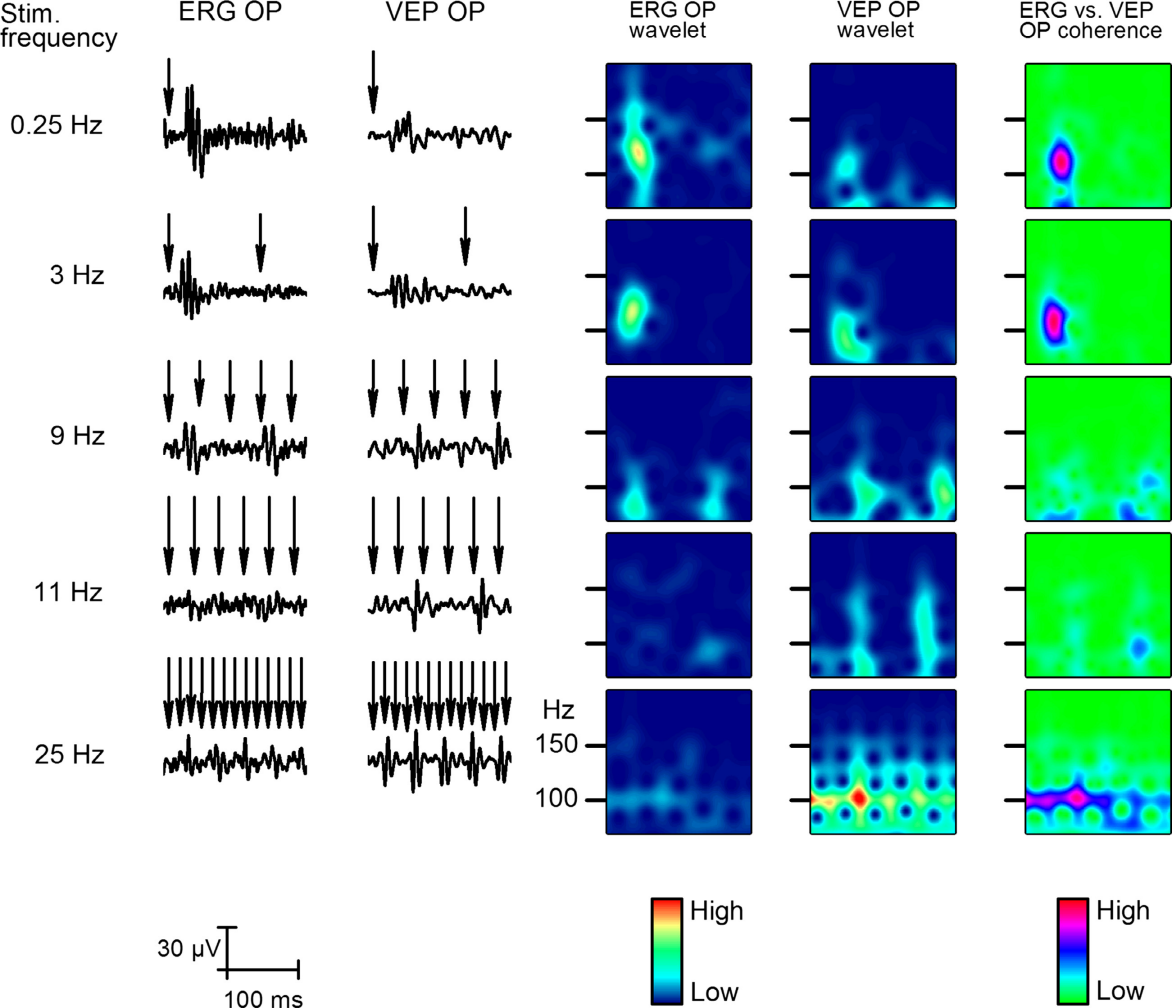
Effect of stimulus frequency on the late response. The two columns on the left show changes in averaged late steady‐state responses (SSR, 1000–1200 msec after stimulus start, *n* = 50) to five different stimulus frequencies from 0.25 to 25 Hz. The next two columns show the wavelet analysis results of late SSR OPs filtered form the retina and the V1. The column on the right indicates coherence of retina and V1 late SSR OPs at different frequencies. The top row is the same as in Figure 6.

To determine the effect of stimulus frequency on the responses recorded at different levels along the visual pathway. We applied flash series for 2 sec, at different stimulus rates. Single flash is modeled with the 0.25 Hz, the 3 Hz is for saccadic eye movement (Schutz et al. 2011), 9–10 Hz to test the starting continuity illusion (Purves et al. 1996) and 25 Hz for the albino rat's critical fusion frequency (Williams et al. 1985) and also because it's very similar to the rate at which theatre movies are filmed. The first 200 msec has been defined as early (Fig. 6) and the 1000–1200 msec period as late stage (Fig. 7) of the response. The first flash (Fig. 6) evokes a response similar to that of the single flash and then the steady‐state response (SSR) gradually develops later above 9 Hz (Fig. 6).

The steady‐state response (SSR) can be examined best in the second half of the stimulation period (Fig. 7). Every visualization method (wavelet, cross‐coherence) shows robust increase in SSR power, and strong coherence among the structures. The sustained coherence was observed near 100 Hz.

Wavelet, cross‐coherence of the retinal response and the V1 response at 9, 11, and 25 Hz stimulus rates changed from stimulus‐locked coherence to a sustained form of coherence exactly which is in line with the albino rat's critical fusion frequency phenomenon.

**Figure 8:**
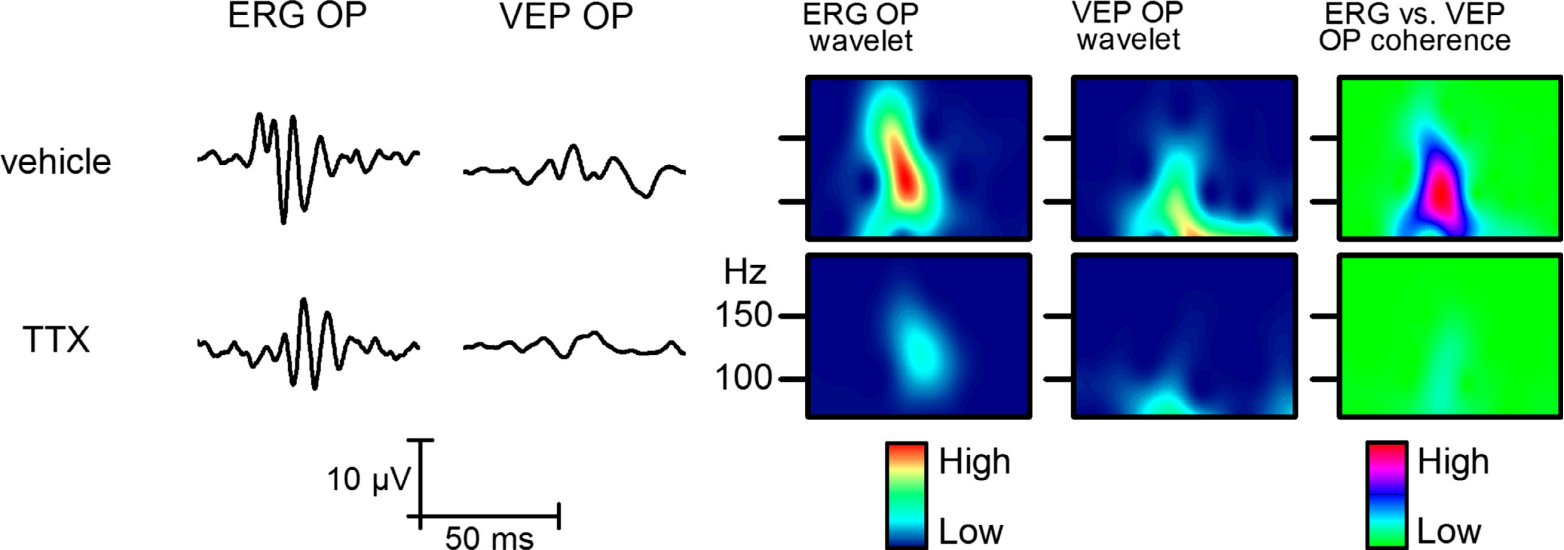
Effect of TTX application to the V1. The two columns on the left show changes in averaged responses (*n* = 50) to administration of TTX onto the V1 cortex. The next two columns show the wavelet plots of OPs filtered form the retina and the V1. The column on the right indicates coherence between the retina and the V1 response OPs.

To demonstrate that the OPs in the V1 are generated by local neurons rather than being volume conducted, we applied 1 μL, 1 μmol/L TTX to the V1 surface (Fig. 8). Electroretinogram and VEP were elicited by single flash of 1 msec duration at 0.25 Hz. Without TTX, both retinal and V1 OPs were present. After applying TTX, the oscillatory components of VEP disappeared and the slow components were reduced suggesting that VEP oscillatory responses were generated in the cortex. The retinogram OPs were detectable in both control and TTX experiments; however, there was a small decrease in the retina OPs after TTX, which could result from the TTX being picked up by the circulatory system.

## Discussion

### Oscillatory potentials support retino‐cortical oscillatory coupling

We proved that the OPs of retinal origin are transferred to the V1 through the optic nerve. Comparison of the peak latencies of OP components in the retina, chiasm, and V1 clearly demonstrate a successive increase in latency times from the retina to the cortex. The OPs have different wave shapes in the retina, chiasm, and V1, which argues against their passive spread. Moreover, at each part of the visual system, the OPs behave similarly when flash luminance, duration, and frequency are changed. At the moment, it is not clear how an oscillatory field potential can spread along a peripheral nerve. In the cortex, the coherence of long‐range gamma synchronization is due to the timed action potential input to the target area driving local field oscillations in the local neuronal circuits at gamma range (Tort et al. 2009). However, it cannot be the case for the chiasm OPs because there are no nerve cells in the chiasm, thus no local circuit exists in this structure. Therefore, the OPs may spread along the optic nerve itself as synchronous local field oscillation on the axon membranes, although there is no clear evidence for this due to the technical difficulties of recording optic nerve field potentials. One could speculate about rhythmic spike activity on the optic nerve but this has not been demonstrated experimentally. Another channel of transmission may be the microtubules that have extremely high dipole characteristics (Schoutens 2005).

The retinal origin of V1 OPs was supported by cortically applied TTX that blocked the VEP OPs but not the retinogram OPs. This suggests that the generation of the VEP OPs is based on the local oscillatory firing of the V1 neurons driven by the retinal input. The TTX‐to‐V1 experiment showed that the VEP OPs require neuronal firing in synchrony with the retinogram OPs, as previously demonstrated by Fries et al. (2002). In conclusion, we found propagation of the OPs on the optic nerve through the optic chiasm but the mechanism of its spreading on the optic nerve is still to be investigated.

### OPs are timing signals carrying little if any information about flash intensity

The photon number in a flash can be varied on different ways. We can change the luminance of the flash by adjusting the electric current driving the LED below the saturation current. LEDs emit photons proportional to the electric current through the crystal that increases exponentially with the current up to the saturation current and further increase in the electric pulse current results in dissipation of the energy excess as heat. Adjusting the luminance of the flash by current on LED, we revealed that at the low end there is some change in the OPs but the OP latency is saturated far under the saturation current of LED crystal. It suggests that the OPs may reflect the flash luminance in a limited range. Also, when the duration of the flash was changed, we observed small changes in the OPs but it was not proportional to the length of time while photons were emitted by the LED. Therefore, we conclude that the OPs rather represent mainly stimulus timing but not intensity. The analogy of OPs and gamma oscillations would suggest the OPs as temporary coupling signals of the visual system that mark the time frame to aid the evaluation of incoming information stream from the retina. Such kind of time‐locked processing of visual information is suggested by the stabilized image experiments by (Ditchburn 1973) and discussed as *packet‐based communication* in the cortex by Luczak et al. (2015).

### OPs reflect the stimulus frequency‐dependent visual processing

In contrast to the stimulus intensity, the OPs change significantly with stimulus frequency or interstimulus interval (ISI). When we delivered two flashes at different interstimulus intervals, we observed that the OPs of the second flash response recover above 200 msec ISI. At shorter intervals, no OPs were observed. In SSR studies, the OPs disappeared for a few hundred milliseconds and recovered, but still have different waveform at 300–500 msec ISI. As the time intervals shift from single flash response to SSR that started at 10 Hz stimulation rate, the flashing light became a faster flickering light without dark sensation between stimuli which is consistent with the finding of Purves et al. (1996). In SSR studies, the recovery of OPs also required a long time shown on wavelet plots. As revealed on coherence analysis plots, the first response OP showed a wide frequency distribution and after the recovery of SSR OPs, continuous gamma frequency coupling at 70–80 Hz developed in correlation with a change in perception form flickering to continuous light stimuli at 25 Hz. This means that the temporary flash onset‐coupled OP shifted to continuous gamma frequency in retina and V1 at the movie frame rate.

## Conclusions

Coherence and wavelet analysis of gamma‐frequency oscillatory potentials in the visual system revealed that the single‐flash OPs are flash onset‐locked oscillations that travel to the V1 via the optic nerve and they evoke the V1 gamma activity. The OPs do not reflect intensity related stimulus parameters but they change with stimulus frequency. When stimulus frequency is increased, the flash onset‐coupled OPs are transformed to continuous gamma. Coherence coupling emerges as the flickering light frequency approaches 25 Hz which is close to the frame rate of a movie. Thus, we suggest a gamma coupling‐based model that is able to switch the visual processing mode from temporary gamma‐coupled, static vision to fluently gamma‐coupled dynamic or movie processing. The static vision mode subserves the binding of all detailed information about features such as texture, shape, color, etc., whereas the dynamic mode merges kinetic information to create fluent movement perception as we handle movie projection. Our freely moving animal model gives a novel tool for studies of the physiological basis of movie and other dynamic visual illusions.

## Conflict of Interest

None declared.
